# Effects of Dietary *Limosilactobacillus fermentum* and *Lacticaseibacillus paracasei* Supplementation on the Intestinal Stem Cell Proliferation, Immunity, and Ileal Microbiota of Broiler Chickens Challenged by Coccidia and *Clostridium perfringens*

**DOI:** 10.3390/ani13243864

**Published:** 2023-12-15

**Authors:** Shuangshuang Guo, Wenfei Tong, Ya Qi, Meihan Jiang, Peng Li, Zhengfan Zhang, Qunbing Hu, Zhuan Song, Binying Ding

**Affiliations:** 1 Engineering Research Center of Feed Protein Resources on Agricultural by-Products, Ministry of Education, Hubei Key Laboratory of Animal Nutrition and Feed Science, Wuhan Polytechnic University, Wuhan 430023, China; guo1shuangshuang@163.com (S.G.); tong1wenfei@163.com (W.T.); qiya18437908067@163.com (Y.Q.); 15943677085@163.com (M.J.); pengli@whpu.edu.cn (P.L.); zhang850820@hotmail.com (Z.Z.); 2 Hubei Horwath Biotechnology Co., Ltd., Xianning 437099, China; huqunbing@163.com; 3 Hunan International Joint Laboratory of Animal Intestinal Ecology and Health, Laboratory of Animal Nutrition and Human Health, Hunan Provincial Key Laboratory of Animal Intestinal Function and Regulation, College of Life Sciences, Hunan Normal University, Changsha 410081, China

**Keywords:** *Limosilactobacillus fermentum*, *Lacticaseibacillus paracasei*, necrotic enteritis, intestinal health, broiler chicken

## Abstract

**Simple Summary:**

Necrotic enteritis (NE) is an important enteric disease in poultry and causes great economic losses to the industry worldwide. *Clostridium perfringens* is a key etiological agent of NE and coccidiosis is a major predisposing factor to NE. With the reduction of antibiotic use in animal production, the outbreak of NE has been increasing in recent years. Probiotics, such as *Lactobacilli*, *Bacillus*, *Bifidobacteria*, and *Enterococcus*, are potentially alternative strategies to mitigate NE in poultry. The protective effects of *Limosilactobacillus fermentum* and *Lacticaseibacillus paracasei* on intestinal barrier function of broilers challenged by coccidia and *C. perfringens* have been reported in our previous publication. The current study concluded that both *Lactobacilli* alleviated the impairment of intestinal stem cell proliferation and immunity in coccidia- and *C. perfringens*-challenged birds via modulating JAK/STAT signaling and reshaping intestinal microflora. It evidenced that *L. fermentum* and *L. paracasei* could be used to prevent or alleviate NE in poultry.

**Abstract:**

This study was conducted to investigate effects of dietary *Limosilactobacillus fermentum* and *Lacticaseibacillus paracasei* supplementation on the intestinal stem cell proliferation, immunity, and ileal microbiota of broiler chickens challenged by coccidia and *Clostridium perfringens*. A total of 336 one-day-old Ross 308 chickens were randomly assigned into four groups. Chickens in the control (CTR) group were fed basal diet, and chickens in the three challenged groups were fed basal diets supplemented with nothing (CCP group), 1.0 × 10^9^ CFU/kg *L. fermentum* (LF_CCP group), and 1.0 × 10^9^ CFU/kg *L. paracasei* (LP_CCP group), respectively. All challenged birds were infected with coccildia on day 9 and *Clostridium perfringens* during days 13–18. The serum and intestinal samples were collected on days 13 and 19. The results showed that *L. fermentum* significantly increased jejunal gene expression of *cdxB* (one of the intestinal stem cell marker genes) on day 13. Additionally, *L. fermentum* significantly up-regulated mRNA levels of *JAK3* and *TYK2* and tended to increase *STAT6* mRNA expression in jejunum on day 19. In the cecal tonsil, both *L. fermentum* and *L. paracasei* decreased mRNA expression of *JAK2* on day 13, and *L. fermentum* down-regulated *JAK1-2*, *STAT1*, and *STAT5-6* gene expressions on day 19. Ileal microbiological analysis showed that coccidial infection increased the *Escherichia*–*Shigella*, *Lactobacillus*, and *Romboutsia* abundance and decreased *Candidatus_Arthromitus* richness on day 13, which were reversed by *Lactobacillus* intervention. Moreover, *Lactobacilli* increased ileal *Lactobacillus* richness on day 19. In conclusion, *Lactobacilli* alleviated the impairment of intestinal stem cell proliferation and immunity in coccidia- and *C. perfringens*-challenged birds via modulating JAK/STAT signaling and reshaping intestinal microflora.

## 1. Introduction

Necrotic enteritis (NE) is an enteric poultry disease that seriously affects profitability in the broiler industry, including clinical and subclinical forms [1,2]. The main characteristics of clinical NE are diarrhea and intestinal necrotic lesions, which lead to high morbidity and mortality in poultry. The subclinical form of NE is mainly characterized by poor growth performance without death or with low mortality [3]. It has been reported that the cost of output losses and control due to NE is approximately 6 billion USD per year [4]. The outbreak of NE is a complex process that includes multiple predisposing factors, among which the presence of pathogenic *Clostridium perfringens* is an important inducement [2,5]. *C. perfringens* is a spore-forming Gram-positive bacterium common in the environment [5]. Nevertheless, *C. perfringens* is a conditional pathogen, and applying it alone to challenge broiler chickens to induce NE is not ideal in some experimental cases due to differences in individual birds or dietary factors. In line with what previous studies reported, we verified that co-infection with coccidia and *C. perfringens* could effectively improve the stability of the necrotic enteritis model in broilers [6,7]. Furthermore, coccidiosis is one of the major predisposing factors contributing to the outbreak of NE under field conditions [4].

Previous findings have revealed that NE infection impairs immune function and causes intestinal damage in broilers [8,9]. Traditional treatments for broiler NE mainly rely on antibiotics. However, due to the ban on the use of antibiotics as feed additives nowadays, it seems to be a feasible alternative strategy to prevent or treat enteritis through nutritional intervention. Presently, plant extracts [10], organic acids [11], oligosaccharide [12], polysaccharides [13,14], and probiotics [15] have been demonstrated to be effective measures to promote animal growth. Probiotics have been reported to stimulate digestion, improve feed efficiency, and increase growth performance by modulating the intestinal microbiota and immune system, inhibiting pathogens, and improving intestinal integrity. Additionally, some strains increase the nutritional value of feed by production of vitamins, exopolysaccharides, and antioxidants [15,16]. A variety of probiotics have been found to exert essential functions for improving growth performance and intestinal health in broilers, mainly including *Bacillus*, *Enterococcus*, *Lactobacillus*, and *Clostridium* [16,17,18,19]. Among that, studies reported that *Limosilactobacillus fermentum* can alleviate intestinal villus damage and increase serum IgM level in broilers challenged with *Salmonella* [20], and relieve *Campylobacter coli* induced inflammatory response by suppressing the secretion of cytokines and modulating lymphocyte subpopulation levels [21]. In addition, Xu et al. demonstrated that *Lacticaseibacillus paracasei* has the ability to enhance growth performance by regulating intestinal microflora in chickens [22]. Moreover, our previous study also found that supplementation with *L. fermentum* and *L. paracasei* enhanced the intestinal health of broilers with NE [7]. However, the underlying mechanism by which *L. fermentum* and *L. paracasei* convey protective effects on broilers is still unclear. 

The occurrence of enteritis is closely related to the impairment of intestinal barrier function, mainly involving the integrity of intestinal structural, inflammatory response, proliferation, and differentiation of intestinal stem cells, and the homeostasis of intestinal microorganisms [23]. Therefore, in the present study, co-infection with coccidia and *C. perfringens* was used to induce NE to further investigate the influence of *L. fermentum* and *L. paracasei* on jejunal stem cell proliferation, immunity, and ileal microbial composition in broilers, which may contribute to gaining a more comprehensive understanding of these probiotics.

## 2. Materials and Methods

### 2.1. Experimental Animals, Diets, and Treatments

All animal procedures used in the present study were approved by the Institutional Animal Care and Use Committee of Wuhan Polytechnic University (Number: WPU202104002). A total of 336 one-day-old chicks (Ross 308) were randomly assigned to 4 groups. Each group contained 6 replicates, while each replicate included 7 males and 7 females. The 4 treatment groups were as follows: the control group (CTR, basal diet); (2) the coccidia and *C*. *perfringens* infected group (CCP, basal diet + coccidia and *C*. *perfringens* infection); (3) *L*. *fermentum*-treated and infected group (LF_CCP, basal diet plus 1.0 × 10^9^ CFU/kg *L*. *fermentum* + coccidia and *C*. *perfringens* infection); and (4) *L. paracasei*-treated and infected group (LP_CCP, basal diet plus 1.0 × 10^9^ CFU/kg *L. paracasei* + coccidia and *C*. *perfringens* infection). The *L. fermentum* (CGMCG No. 1.2029) was obtained from the China General Microbiological Culture Collection Center (CGMCG, Beijing, China). The *L. paracasei* was obtained from the State Key Laboratory of Agricultural Microbiology, Huzhong Agricultural University. Both *Lactobacilli* were cultured in Mann–Rogosa–Sharpe broth and then incorporated in the diets, which were formulated once a week. The corn–soybean meal basal diets were formulated according to the recommendation of the National Research Council (NRC, 1994). The feed ingredient composition and nutritional level of the basal diet are reported in Table 1. The trial period was 19 days.

On day 9 of the trial, birds in the CCP, LF_CCP, and LP_CCP groups were treated with attenuated coccidial vaccine, and each bird was inoculated with 33,000 ± 3300 spores (30-fold of recommended dose). Birds in the CTR group were mock-infected with an equal volume of saline. The coccidial vaccine used in the present study consisted of 1 × 10^5^ oocysts of *E*. *acervuline* strain PAHY, and 5 × 10^4^ oocysts of *E*. *tenella* strain PTMZ, *E*. *maxima* strain PMHY, as well as *E*. *necatrix* strain PNHZ, which were purchased from Foshan Zhengdian Biotechnology Co., Ltd. (Foshan, Guangdong, China). From days 13 to 18, broilers in the CCP, LF_CCP, and LP_CCP groups were infected with type A *C*. *perfringens* via feed. A total of 64 mL of *C*. *perfringens* broth, containing *C*. *perfringens* 1 × 10^8^ CFU/mL, was well mixed with 1.2 kg feed in each group. An equal volume of sterile broth was incorporated into the diet in the CTR group. The basal diets were withdrawn for 3 h before infection and the contaminated feeds containing *C. perfringens* were consumed within 2 h. Type A *C*. *perfringens* (CVCC2030) was obtained from the China Veterinary Microbial Culture Collection and Management Center (Beijing, China). All birds were reared in wire cages and allowed to access feed and water freely. Room temperature was manually controlled. A 24 h light was provided throughout the trial.

### 2.2. Sample Collection

On days 13 and 19, two chickens per replicate were randomly selected to collect blood from wing veins and euthanized by cervical dislocation, and then slaughtered for sample collection. The serum was obtained by centrifugation at 4 °C, 3000× *g* for 15 min, and stored at −80 °C for further analysis. Jejunal mucosa was scraped from about 10 cm of the jejunal segment and stored at −80 °C for RNA isolation. Cecal tonsils were also rapidly separated for total RNA isolation. Digesta from the ileum was collected and stored at −80 °C for microbial composition analysis.

### 2.3. Serum IgA and Cytokine Levels

Levels of IgA, IL-1β, IL-8, IL-10, and IFN-γ in serum were determined using an enzyme-linked immunosorbent assay performed by Beijing Kangjia Hongyuan Biotechnology Co., Ltd. (Beijing, China).

### 2.4. Quantitative Real-Time PCR

The extraction of total RNA from jejunal mucosa and the cecal tonsil, preparation of cDNA (the reverse transcription of RNA), and RT-qPCR were performed as previously described [24]. Briefly, total RNA was obtained using the TRIzol reagent (Takara, Dalian, China) according to the manufacturer’s instructions. The concentration of RNA and its OD260/280 value was quantified using the NanoDrop^®^ ND-2000 UV-VIS spectrophotometer (Thermo Scientific, Wilmington, DE, USA). The cDNA was synthesized from 1 μg RNA using a PrimeScript^®^ RT reagent Kit with gDNA Eraser (Takara, Dalian, China) as the manufacturer’s protocol. An ABI-Prism 7500 sequence detection system (Applied Biosystems, Foster City, CA, USA) was used to perform RT-qPCR procedures. The relative mRNA level of each gene was calculated by the 2^−ΔΔCt^ method and was normalized by *β-actin*. Primer sequences used in the present study are shown in Table 2. Primers were synthesized by Sangon Biotech (Shanghai) Co., Ltd. (Shanghai, China).

### 2.5. Ileal Microbiota Analysis

The ileal microbial DNA extraction, amplification, and sequencing were performed by Novogene Technology Co., Ltd. (Beijing, China). Briefly, bacterial DNA was extracted from the ileal digesta using a QIAamp DNA Stool Mini Kit (Qiagen Inc., Valencia, CA, USA). The integrity of DNA was verified by agarose gel electrophoresis. The qualified DNA was used as a template for PCR amplification with 341F and 805R primers (5′-CCTACGGGNBGCASCAG-3′ and 5′-GACTACNVGGGTATCAATCC-3′) targeting the variable V3–V4 gene region. Purified PCR amplification products were used to construct sequencing libraries on a Hiseq PE250 (Illumina, CA, USA). Paired-end reads were demultiplexed and quality-filtered by Trimmomatic (version 0.36) and then merged by Flash (version 1.2.11). The sequences were clustered into operational taxonomic units (OTUs) with 97% similarity using UPARSE (version 7.1). The taxonomy of each OTU representative sequence was analyzed by SILVA database (v138). Alpha diversity analysis, including Shannon and relative abundance of bacteria, was performed with Quantitative Insights into Microbial Ecology (QIIME). The different biomarker bacteria between groups were determined using the linear discriminant analysis effect size (LEfSe) method based on the linear discriminant analysis (LDA) values.

### 2.6. Statistical Analysis

Data in figures are displayed as means ± SEM and data in tables are expressed as mean and pooled SEM. The data of serum parameters, gene expression, and microbial alpha diversity were analyzed by one-way ANOVA with SPSS 26.0 statistical software (SPSS, Inc., Chicago, IL, USA). The Tukey multiple comparison was performed when significant differences were noticed among groups. Graphs were created using GraphPad Prism 8.0 software (GraphPad Software, Inc., San Diego, CA, USA). *p* < 0.05 was considered significantly different between groups.

## 3. Results

### 3.1. Expression of Genes Involving Stem Cell Proliferation in Jejunum

**Figure 1:**
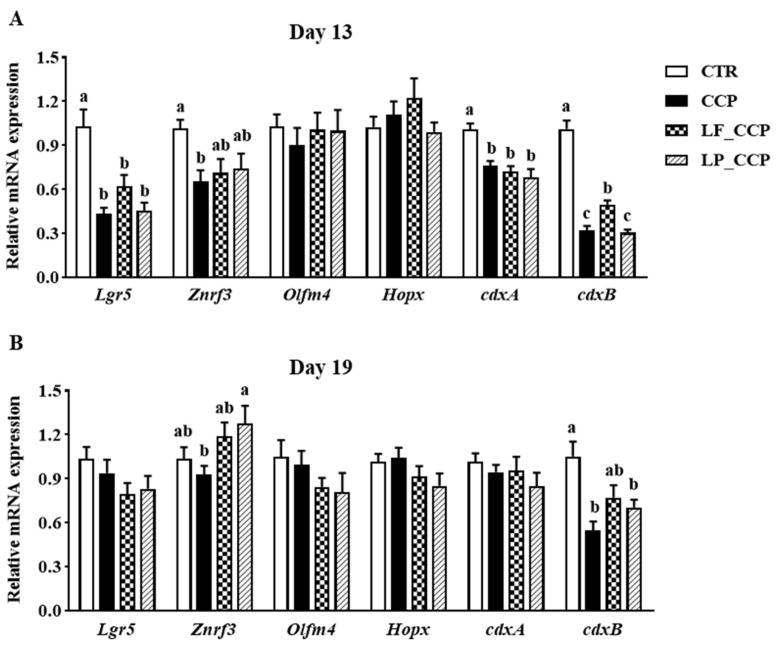
Effects of dietary *L. fermentum* and *L. paracasei* supplementation on mRNA expression of genes related to the proliferation of intestinal stem cells in broiler chickens challenged with coccidia and *C. perfringens* on days 13 (**A**) and 19 (**B**). Data are presented as means ± SEM (*n* = 12). Bars with different letters differ significantly (*p* < 0.05), and bars with the same letters or without letters did not differ significantly (*p* > 0.05). CTR, unchallenged group; CCP, coccidia- and *Clostridium perfringens*-challenged group; LF_CCP, challenged group with dietary supplementation of *Limosilactobacillus fermentum*; LP_CCP, challenged group with dietary supplementation of *Lacticaseibacillus paracasei*. *Lgr5*, leucine-rich repeat containing G protein-coupled receptor 5; *Znrf3*, zinc and ring finger 3; *Olfm4*, olfactomedin 4; *Hopx*, homeodomain-only protein X; *cdx*, caudal-type homeobox.

As shown in Figure 1, compared with the CTR group, coccidia and *C. perfringens* infection significantly reduced mRNA levels of leucine-rich repeat containing G protein-coupled receptor 5 (*Lgr5*), zinc and ring finger 3 (*Znrf3*), caudal-type homeobox 1 (*cdxA*), and *cdxB* in jejunum on day 13, as well as *cdxB* gene expression in jejunum on day 19 (*p* < 0.05). In addition, the LF_CCP group alleviated the decreased expression of *cdxB* in challenged broilers on day 13 and tended to alleviate it on day 19 (*p* < 0.05), and two *Lactobacilli* had tendency to attenuate the down-regulation of *Znrf3* mRNA level on day 13. Compared with the CCP group on day 19, the LF_CCP group showed a tendency to elevate *Znrf3* gene expression, and the LP_CCP group significantly up-regulated *Znrf3* gene expression (*p* < 0.05).

### 3.2. Serum IgA and Cytokine Level

**Figure 2:**
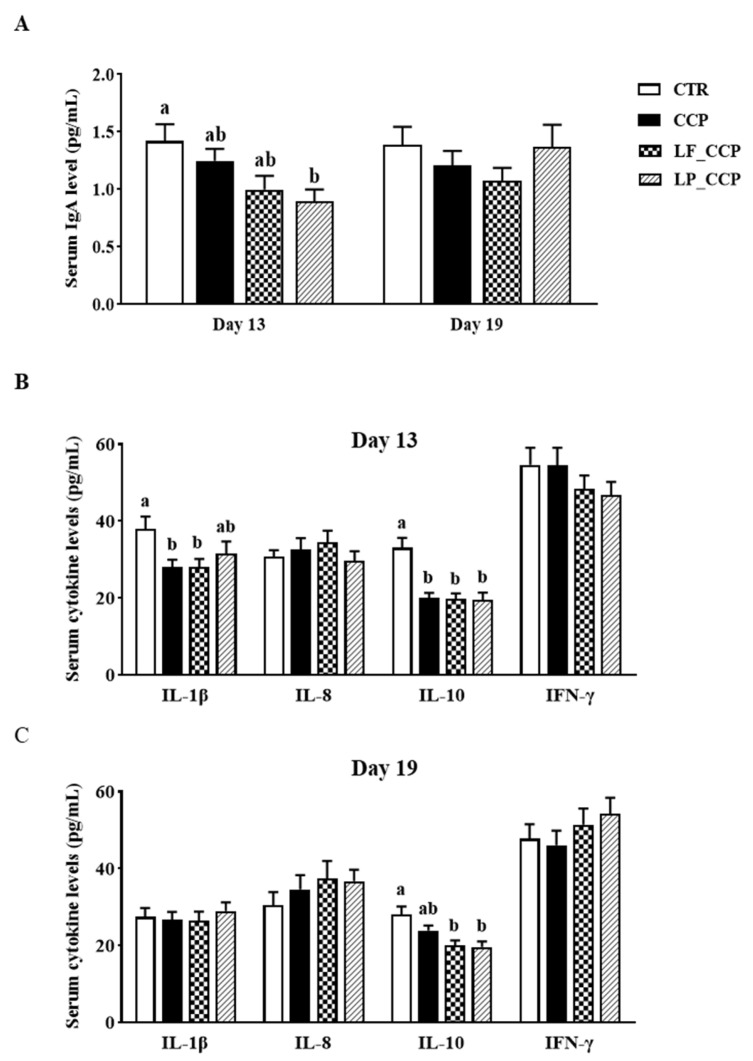
Effects of dietary *L. fermentum* and *L. paracasei* supplementation on serum IgA (**A**) and cytokine (**B**,**C**) levels in broiler chickens challenged with coccidia and *C. perfringens* on days 13 and 19. Data are presented as means ± SEM (*n* = 12). Bars with different letters differ significantly (*p* < 0.05), and bars with the same letters or without letters did not differ significantly (*p* > 0.05). CTR, unchallenged group; CCP, coccidia and *Clostridium perfringens*-challenged group; LF_CCP, challenged group with dietary supplementation of *Limosilactobacillus fermentum*; LP_CCP, challenged group with dietary supplementation of *Lacticaseibacillus paracasei*. IL, interleukin; IFN-γ, interferon-γ.

As presented in Figure 2, compared with the CTR group, the LP_CCP group significantly decreased serum IgA level on day 13, (*p* < 0.05), while the CCP and LF_CCP groups did not significantly affect it (*p* > 0.05). There were no significant differences of serum IgA levels among the groups on day 19 (*p* > 0.05). The CCP and LF_CCP groups significantly decreased serum IL-1β and IL-10 concentrations in broilers when compared with the CTR group on day 13 (*p* < 0.05), and the LP_CCP group reduced serum IL-10 level (*p* < 0.05). Both *L. fermentum* and *L. paracasei* supplementation decreased serum IL-10 levels in coccidia- and *C. perfringens*-infected broilers compared with their control counterparts on day 19 (*p* < 0.05).

### 3.3. Immune-Related Gene Expression in Jejunum

**Figure 3:**
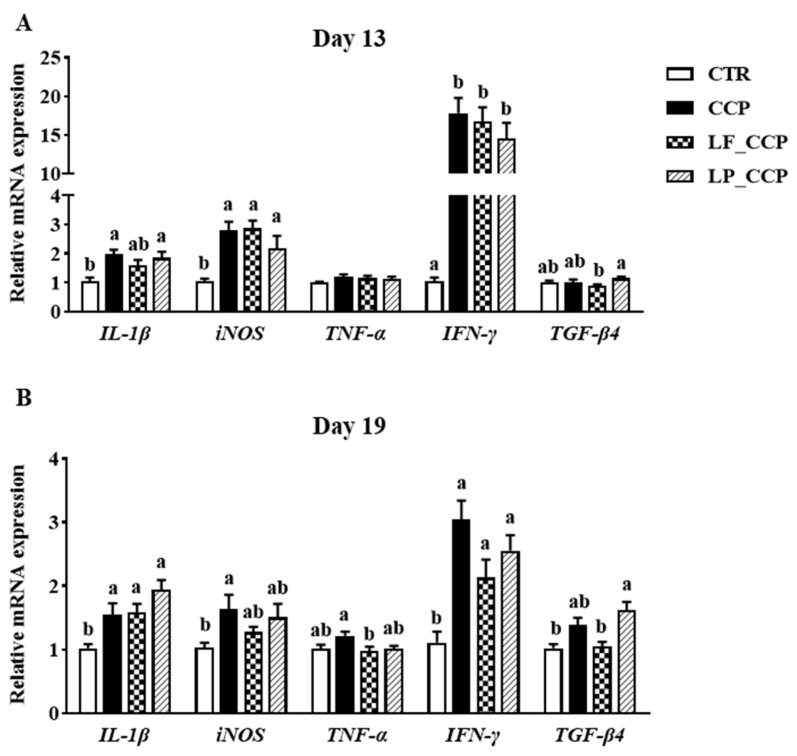
Effects of dietary *L. fermentum* and *L. paracasei* supplementation on jejunal cytokine mRNA expression in broiler chickens challenged with coccidia and *C. perfringens* on days 13 (**A**) and 19 (**B**). Data are presented as means ± SEM (*n* = 12). Bars with different letters differ significantly (*p* < 0.05), and bars with the same letters or without letters did not differ significantly (*p* > 0.05). CTR, unchallenged group; CCP, coccidia- and *Clostridium perfringens*-challenged group; LF_CCP, challenged group with dietary supplementation of *Limosilactobacillus fermentum*; LP_CCP, challenged group with dietary supplementation of *Lacticaseibacillus paracasei*. *IL*, interleukin; *iNOS*, inducible nitric oxide synthase; *TNF-α*, tumor necrosis factor-α; *IFN-γ*, interferon-γ; *TGF-β4*, transforming growth factor-β4.

As displayed in Figure 3, coccidia and *C. perfringens* challenge remarkably increased mRNA levels of *IL-1β*, *iNOS*, and *IFN-γ* in the jejunum of broilers both on days 13 and 19 (*p* < 0.05). Compared with the challenged group, *L. fermentum* or *L. paracasei* addition tended to down-regulate jejunal *iNOS* expression on day 19; moreover, L. fermentum significantly decreased *TNF-α* (*p* < 0.05) while alleviating the increase in *TGF-β4* expression.

### 3.4. Immune-Related Gene Expression in Cecal Tonsil

**Figure 4:**
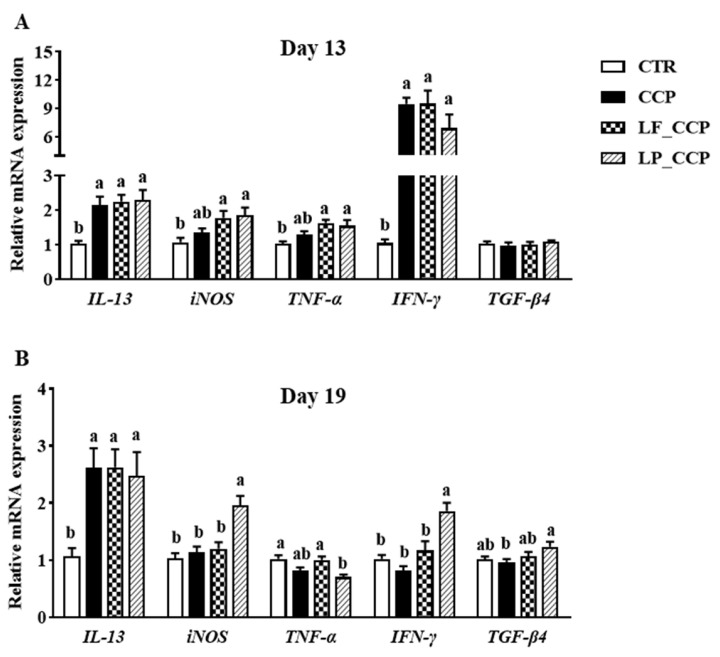
Effects of dietary *L. fermentum* and *L. paracasei* supplementation on cytokine mRNA expression in the cecal tonsil of broiler chickens challenged with coccidia and *C. perfringens* on days 13 (**A**) and 19 (**B**). Data are presented as means ± SEM (*n* = 12). Bars with different letters differ significantly (*p* < 0.05), and bars with the same letters or without letters did not differ significantly (*p* > 0.05). CTR, unchallenged group; CCP, coccidia- and *Clostridium perfringens*-challenged group; LF_CCP, challenged group with dietary supplementation of *Limosilactobacillus fermentum*; LP_CCP, challenged group with dietary supplementation of *Lacticaseibacillus paracasei*. *IL*, interleukin; *iNOS*, inducible nitric oxide synthase; *TNF-α*, tumor necrosis factor-α; *IFN-γ*, interferon-γ; *TGF-β4*, transforming growth factor-β4.

On day 13, higher mRNA levels of *IL-13* and *IFN-γ* in the cecal tonsil were detected in the CCP group (*p* < 0.05, Figure 4A). On day 19, coccidia and *C. perfringens* challenge also increased the IL-13 mRNA level when compared with the CTR group (*p* < 0.05, Figure 4B). Neither *L. fermentum* nor *L. paracasei* attenuated the up-regulation of these genes. On the contrary, LP_CCP group had the highest transcript level of *iNOS* and *IFN-γ* in the cecal tonsil among groups on day 19 (*p* < 0.05).

### 3.5. Expression of Key Genes in the JAK/STAT Signaling Pathway in the Jejunum

**Figure 5:**
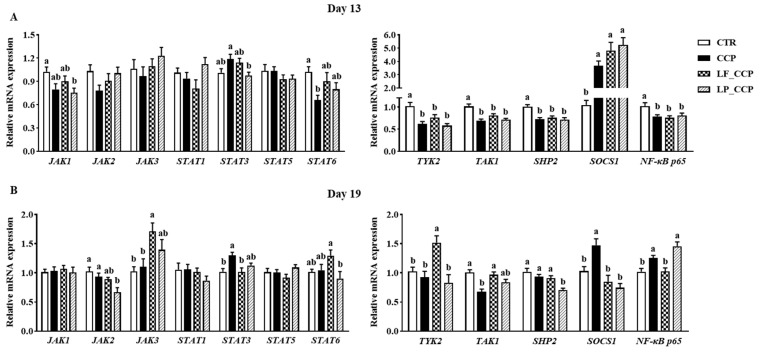
Effects of dietary *L. fermentum* and *L. paracasei* supplementation on mRNA expression of genes related to JAK/STAT and NF-κB signaling in the jejunum of broiler chickens challenged with coccidia and *C. perfringens* on days 13 (**A**) and 19 (**B**). Data are presented as means ± SEM (*n* = 12). Bars with different letters differ significantly (*p* < 0.05), and bars with the same letters or without letters did not differ significantly (*p* > 0.05). CTR, unchallenged group; CCP, coccidia- and *Clostridium perfringens*-challenged group; LF_CCP, challenged group with dietary supplementation of *Limosilactobacillus fermentum*; LP_CCP, challenged group with dietary supplementation of *Lacticaseibacillus paracasei*. *JAK*, Janus-activated kinase; *STAT*, signal transducer and activator of transcription; *TYK2*, tyrosine kinase 2; *TAK1*, transforming growth factor kinase 1; *SHP2*, src homology-2 domain-containing protein tyrosine phosphatase 2; *SOCS1*, suppressors of cytokine-signaling 1; *NF-κB*, nuclear factor-κB.

On day 13, as presented in Figure 5A, the CCP group had higher suppressors of cytokine-signaling 1 (*SOCS1*) mRNA levels and lower mRNA levels of *STAT6*, tyrosine kinase (*TYK2*), transforming growth factor kinase 1 (*TAK1*), Src homology-2 domain-containing protein tyrosine phosphatase 2 (*SHP2*), and *NF-κB p65* than that in the CTR group (*p* < 0.05). The LP_CCP group alleviated the increase in STAT3 in challenged broilers. On day 19, compared with the CTR group, coccidia and *C. perfringens* challenge increased mRNA levels of *STAT3*, suppressors of cytokine-signaling 1 (*SOCS1*), and *NF-κB p65,* and decreased *TAK1* expression in the jejunum (*p* < 0.05, Figure 5B). Meanwhile, compared with the CCP group, *L. fermentum* treatment reversed the gene expression of *STAT3*, *SOCS1*, *NF-κB p65* and *TAK1*, and raised the gene expression of *JAK3* and *TYK2* on day 19 (*p* < 0.05).

### 3.6. Expression of Key Genes in the JAK/STAT Signaling Pathway in the Cecal Tonsil

**Figure 6:**
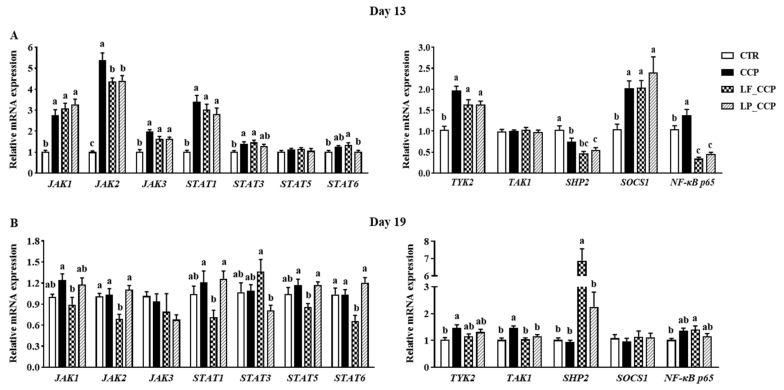
Effects of dietary *L. fermentum* and *L. paracasei* supplementation on mRNA expression of genes related to JAK/STAT and NF-κB signaling in the cecal tonsil of broiler chickens challenged with coccidia and *C. perfringens* on days 13 (**A**) and 19 (**B**). Data are presented as means ± SEM (*n* = 12). Bars with different letters differ significantly (*p* < 0.05), and bars with the same letters or without letters did not differ significantly (*p* > 0.05). Different letters indicate significant differences (*p* < 0.05) between groups. CTR, unchallenged group; CCP, coccidia- and *Clostridium perfringens*-challenged group; LF_CCP, challenged group with dietary supplementation of *Limosilactobacillus fermentum*; LP_CCP, challenged group with dietary supplementation of *Lacticaseibacillus paracasei*. *JAK*, Janus-activated kinase; *STAT*, signal transducer and activator of transcription; *TYK2*, tyrosine kinase 2; *TAK1*, transforming growth factor kinase 1; *SHP2*, src homology-2 domain-containing protein tyrosine phosphatase 2; *SOCS1*, suppressors of cytokine-signaling 1; *NF-κB*, nuclear factor-κB.

On day 13, compared with the CTR group, the CCP group up-regulated mRNA expression of *JAK1*-*3*, *STAT1*, *STAT3*, *TYK2*, *SOCS1*, and *NF-κB p65* in the cecal tonsil, while it down-regulated *SHP2* expression (*p* < 0.05, Figure 6A). Importantly, dietary *L. fermentum* and *L. paracasei* reversed the gene expression of *JAK2* and *NF-κB p65* (*p* < 0.05). On day 19, coccidia and *C. perfringens* infection significantly up-regulated mRNA levels of *TYK2* and *TAK1* (*p* < 0.05, Figure 6B). Intriguingly, the LF_CCP group had the lowest transcriptional levels of *JAK1*, *JAK2*, *STAT1*, *STAT5,* and *STAT6* in the cecal tonsil among groups, whereas it sharply up-regulated the mRNA level of *SHP2* (*p* < 0.05).

### 3.7. Alpha Diversity of Microbiota in Ileum

As shown in Table 3, on day 13, the alpha diversity of ileal microbiota was not significantly affected by coccidia infection compared with the CCP group, and dietary *L. fermentum* significantly increased the Shannon and Simpson indices (*p* < 0.05), and *L. paracasei* tended to increase the Shannon and Simpson indices in challenged broilers. On day 19, the Chao1 and Ace indices in the CCP group were higher than that in the CTR group, although this did not achieve statistical significance. At this time point, compared with the CCP group, dietary *L. paracasei* significantly decreased the Chao1 and Ace indices (*p* < 0.05).

### 3.8. Bacterial Composition at Phylum Level in Ileum

**Figure 7:**
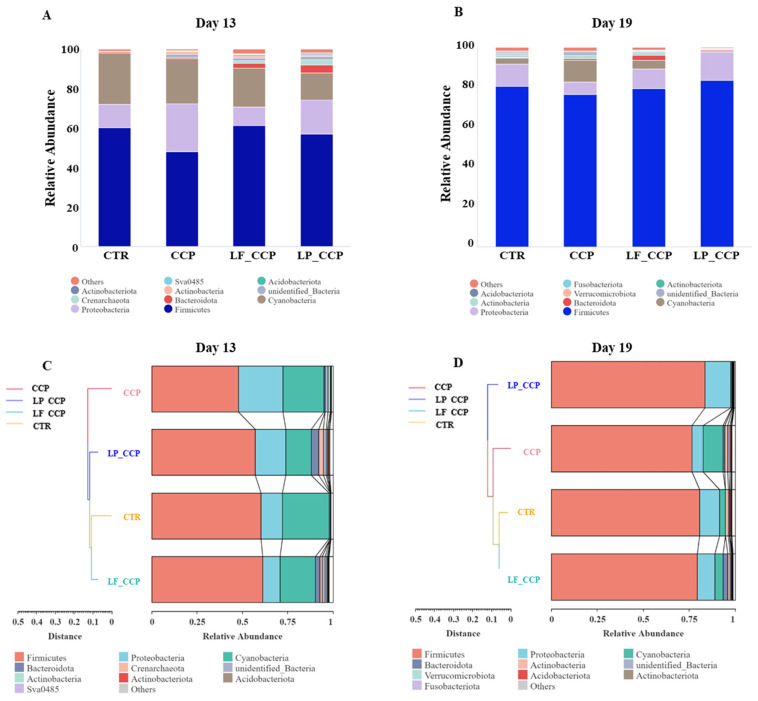
Effects of dietary *L. fermentum* and *L. paracasei* supplementation on ileal microbial composition in broiler chickens challenged with coccidia and *C. perfringens* on days 13 and 19. Relative abundance of microbiota at the phylum level on days 13 (**A**) and 19 (**B**); Cluster analysis of microbial composition on days 13 (**C**) and 19 (**D**). Data were derived from 8 chickens in each group. CTR, unchallenged group; CCP, coccidia- and *Clostridium perfringens*-challenged group; LF_CCP, challenged group with dietary supplementation of *Limosilactobacillus fermentum*; LP_CCP, challenged group with dietary supplementation of *Lacticaseibacillus paracasei*.

On day 13, compared with the control group, the relative abundance of Firmicute was decreased by coccidia infection at the phylum level, whereas the abundance of Proteobacteria was increased (Figure 7A). Compared with the CCP group, dietary *L. fermentum* and *L. paracasei* reversed the abundance of Firmicute and Proteobacteria in ileum of challenged broilers, and both of them enhanced the Bacteroidota abundance. At 19 days of age, coccidia and *C. perfringens* infection reduced the Proteobacteria abundance and increased the Cyanobacteria abundance when compared with the CTR group, which was reversed by dietary supplementation with *L. fermentum* and *L. paracasei* (Figure 7B). Bacteroidota was most abundant in LF_CCP group. Specially, the results of cluster analysis showed that the microbial composition of LF_CCP group was more similar to the CTR group both on days 13 and 19 at the phylum level (Figure 7C,D).

### 3.9. Bacterial Composition at Genus Level in Ileum

**Figure 8:**
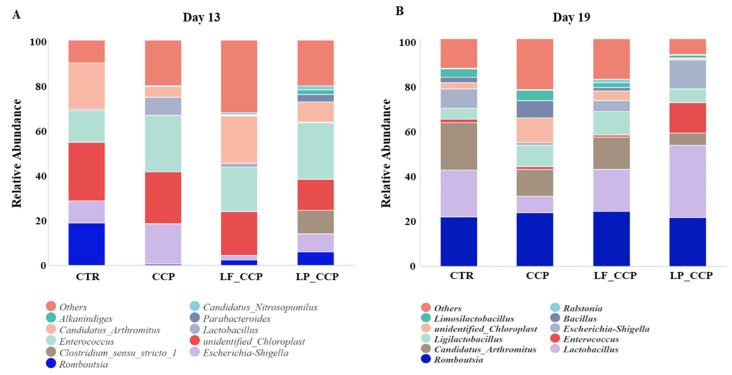
Relative abundance of ileal microbiota at the genus level on days 13 (**A**) and 19 (**B**). Data were derived from 8 chickens in each group. CTR, unchallenged group; CCP, coccidia and *Clostridium perfringens*-challenged group; LF_CCP, challenged group with dietary supplementation of *Limosilactobacillus fermentum*; LP_CCP, challenged group with dietary supplementation of *Lacticaseibacillus paracasei*.

As shown in Figure 8A, at 13 days of age, compared with the control group, *Escherichia–Shigella*, *Enterococcus*, and *Lactobacillus* were highly enriched in the CCP group, while the abundance of *Romboutsia*, *unidentified_Chloroplast*, and *Candidatus_Arthromitus* was decreased. Contrarily, dietary *L. fermentum* and *L. paracasei* diminished the richness of *Escherichia–Shigella* and *Lactobacillus* and enhanced the abundance of *Candidatus_Arthromitus*. Figure 8B depicts the ileal bacterial composition at the genus level on day 19. Compared with the control group, coccidia and *C. perfringens* challenge increased the abundance of *Ligilactobacillus*, *unidentified_Chloroplast*, and *Bacillus*, but decreased *Lactobacillus*, *Escherichia–Shigella*, and *Candidatus_Arthromitus* abundance. Compared with the CCP group, two lactobacillus strains increased the abundance of *Lactobacillus* and *Escherichia–Shigella* and reduced the abundance of *unidentified_Chloroplast* and *Bacillus.* Interesting, dietary *L. fermentum* increased the *Ralstonia* abundance and *L. paracasei* increased the *Enterococcus* abundance.

### 3.10. LEfSe Analysis for Biomarkers

**Figure 9:**
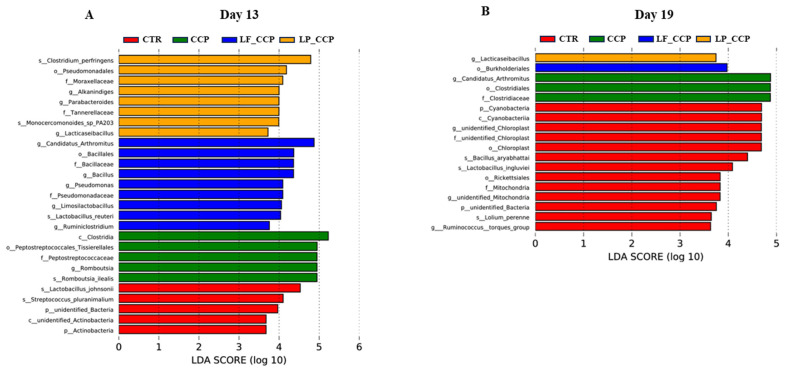
LefSe analysis of different phylotypes between groups on days 13 (**A**) and 19 (**B**). Data were derived from 8 chickens in each group. CTR, unchallenged group; CCP, coccidia- and Clostridium perfringens-challenged group; LF_CCP, challenged group with dietary supplementation of Limosilactobacillus fermentum; LP_CCP, challenged group with dietary supplementation of Lacticaseibacillus paracasei.

LefSe analysis can be used to analyze the statistically different biomarker bacteria between groups. As Figure 9A shows, on day 13, the microbiota of birds in the CCP group were enriched with *Romboutsia.* The *Candidatus_Arthromitus* and *Bacillus* were more abundant in the LF_CCP group, and the *Alkanindiges* and *Parabacteroides* genus were more prevalent in the LP_CCP group. Interestingly, on day 19, the *Candidatus_Arthromitus* was a biomarker genus in the CCP group; meanwhile, the LP_CCP group had a greater abundance of *Lacticaseibacillus* (Figure 9B).

## 4. Discussion

The NE can decrease the growth performance of poultry by impairing intestinal integrity. In the post-antibiotic era, probiotics are one of the effective strategies to protect poultry from NE for its potential growth-promoting property, enhancement of intestinal health, and modulation of immune function [26,27]. The previous and current results showed successful establishment of the coccidia and *C*. *perfringens* co-challenge model, as reflected by decreased average daily gain and average feed intake and increased intestinal lesion score in challenged birds [7]. Importantly, we previously found that *L. fermentum* and *L. paracasei* improved intestinal integrity and barrier function, although they did not alleviate the decrease in growth performance [7]. The homeostasis of intestinal stem cell proliferation, immune function, and microbial composition are all closely correlated to the integrity of the intestine. Herein, the regulatory effects of *L. fermentum* and *L. paracasei* on these intestinal-health-related factors were further explored in the present study.

Cellular losses due to either natural cellular attrition or injury within different tissues are persistently replenished by stem cells to meet the homeostatic need or regenerative demand [28,29]. Intestinal stem cells (ISCs), which are located in the proliferative intestinal crypt, are responsible for the replenishment of intestinal epithelial cells. The self-renewal ability of ISCs further determines the integrity of the epithelial barrier and the rapid repairment of the intestinal epithelium from injury [30,31]. At present, multiple biomarkers of ISCs in chickens have been identified by lineage tracing of candidate quiescent ISCs populations, such as Lgr5, Olfm4, Znrf3, Bmi1, Hopx, cdxA, and cdxB [30,32,33]. Lgr5 is the first discovered intestinal stem cell marker gene, and Lgr5^+^ stem cells have strong self-renewal ability and can differentiate into various types of intestinal cells [32]. The Wnt/β-catenin pathway is a key indicator for the proliferation and maintenance of ISCs and is tightly regulated by E3 ubiquitin ligases Rnf43 and Znrf3, which target Wnt receptors for degradation. Therefore, the normal expression of Znrf3 can control the excessive proliferation of stem cells [34]. CdxA and cdxB homeobox genes, characteristic of proliferating epithelial cells, belong to caudal family transcription factors, and are expressed both in chicken embryos and chicks. The role of these genes in the chicken is not fully understood, but they may be involved in the maturation of the intestine [33]. The decreased expression of Lgr5 and other related stem cell marker genes in the present study revealed that the coccidia and *C. perfringens* infection caused the intestinal injury of broilers and therefore adversely affected the development of ISCs. Importantly, the up-regulated *Znrf3* and *cdxB* gene expression in the *L. fermentum*- and *L. paracasei*-supplemented group highlighted the beneficial effect of probiotics on intestinal stem cell proliferation. Similarly, several studies reported that probiotics (1.0 × 10^8^ CFU/d) could stimulate the proliferation of ISCs in chickens [35,36,37]. In addition, an interesting study showed that *Lactobacillus reuteri D8* (1.0 × 10^4^ CFU/well) could stimulate ISC proliferation through the phosphorylation of STAT3 activated by IL-22 to protect the mucosal barrier in mice induced by dextran sodium sulfate [38]. The present study demonstrated that dietary *L. fermentum* also activated JAK/STAT signaling pathway on day 19, which might contribute to the promotion of ISC proliferation and thereby improve intestinal health of broilers.

Inflammation is a paramount part of the body’s first line of defense against pathogen invasion and plays a crucial role in tissue repairment and regeneration. Increased secretion of pro-inflammatory cytokines is usually accompanied with the occurrence of inflammatory response, such as IL-6, IL-8, IL-1β, and so on [39]. To our surprise, coccidia and *C. perfringens* infection did not evoke a sharp increase in IgA and cytokine production in serum. One possible explanation may be that the systemic inflammatory response of broilers was in the recovery phase on day 13 or the intestinal local inflammation was not strong enough to alter systemic inflammation on day 19. Nevertheless, we found that transcriptional levels of *IL-1β*, *iNOS,* and *IFN-γ* in the jejunum were up-regulated by coccidia and *C. perfringens* infection on both days 13 and 19, indicating strong inflammatory responses in the jejunum of birds. Moreover, consistent with previous studies [20,24], our results showed that *L. fermentum* treatment suppressed *iNOS*, *TNF-α,* and *TGF-β4* expression, which suggested that *L. fermentum* alleviated intestinal inflammatory responses in challenged broilers.

In particular, the prominent rise of *IFN-γ* expression in the jejunum after infection drew our attention. It is well known that IFNs belong to the secreted cytokines that are important regulators of immunity for modulating host defense against pathogenic infection. Moreover, IFNs can regulate the expression of related genes by binding to specific cell-surface receptors and then activating the JAK/STAT signaling pathway [40,41]. A previous study has shown that necrotic enteritis in broilers induced by *C. perfringens* was accompanied by activation of the JAK/STAT pathway [42]. Therefore, we also examined the expression of key genes in the JAK/STAT pathway in the jejunum. Of note, several negative feedback regulators of the JAK/STAT pathway have been identified, mainly including SOCSs, protein inhibitors of activated STAT (PIASs), and protein tyrosine phosphatases (PTPs) [43]. Our results showed that coccidia infection inhibited jejunal JAK/STAT signaling on day 13, reflected by decreased gene expression of *STAT6*, *TYK2*, *TAK1*, and *SHP2*. Consistently, the mRNA level of *SOCS1* was strongly elevated by coccidial infection. The strong inhibition of the JAK/STAT pathway by coccidial infection during the inflammation recovery stage might benefit the maintenance of intestinal homeostasis. As for day 19, *C. perfringens* infection significantly increased *STAT3* expression, but the *SOCS1* expression was also raised. Therefore, it was difficult to distinguish the state of JAK/STAT signaling pathway due to *C. perfringens* challenge. Unexpectedly, *L. fermentum* increased the mRNA abundance of *JAK3*, *STAT6*, and *TYK2*, and consistently decreased the *SOCS1* expression, indicating that the JAK/STAT signaling was greatly activated. This is consistent with the slight attenuation of jejunal inflammation and ISC proliferation by *L. fermentum* supplementation.

The cecal tonsil is the largest intestinal lymphoid tissue in poultry and its indispensable immune function indirectly contributes to the integrity of the intestine [44]. Dietary *L. fermentum* and *L. paracasei* failed to alleviate the up-regulation of cytokines in cecal tonsil. However, both dietary *L. fermentum* and *L. paracasei* reduced the gene expression of *NF-κB p65* on day 13, and *L. fermentum* generally inhibited expression of genes involving in JAK/STAT pathway in the cecal tonsil on day 19. Again, these results suggested that two *Lactobacilli* could alleviate intestinal inflammation via the JAK/STAT signaling pathway, which was concordant with previous findings that probiotics can promote gut health by providing a more stable immune homeostasis in the cecal tonsil [45]. It is worth noting that the different effects of *Lactobacilli* on the immunity of jejunum and cecal tonsil in our present study, especially on the regulation of JAK/STAT signaling, may indicate the tissue-specific immune-modulatory effects.

It has been accepted that intestinal microbiota could regulate intestinal barrier and immune maturation, which contributes to the homeostasis of the intestine and the resistance to pathogens [46]. Our results showed that dietary *L. fermentum* increased the Shannon and Simpson indices of ileal microbes in birds at the age of 13 days, but *L. paracasei*-impaired Chao1 and Ace indices on day 19 compared with the CCP group, which suggested that microbial diversity decreased in the LP_CCP group. Partly in accordance with our study, Li et al. [47] also found that the richness of the ileal bacterial community of chickens was reduced by *Lactobacillus acidophilus*. One possible assumption was that *L. fermentum* and *L. paracasei* reduced the richness of ileal microorganisms through promoting the proliferation of dominant flora. At the phylum level, consistent with previous reports [24,48], we found that Firmicutes, Cyanobacteria, Proteobacteria, and Bacteroidetes were the four predominant bacteria in the ileum of broilers. Compared with unchallenged chickens, the abundance of Firmicutes was decreased and Proteobacteria abundance was enhanced by coccidia infection, which was reversely changed by *L. fermentum* and *L. paracasei* on day 13. Moreover, the Cyanobacteria phylum, which can synthesize toxic secondary metabolites to impair health on animals [49,50], was rich in challenged broilers and was also inversely decreased by *L. fermentum* and *L. paracasei* administration on day 19. It was reported that a decrease in Firmicutes and an increase in Proteobacteria was correlated with the severity of NE in birds [24,51], and the ratio of Firmicutes/Bacteroides was associated with host health and growth performance [52]. The abundant Firmicutes and less abundant Cyanobacteria in lactobacillus-treated groups may be conducive to the homeostasis of ileal microbial composition. However, *C. perfringens* infection had no significant effect on the abundance of Firmicutes and even decreased the abundance of Proteobacteria on day 19. We speculated that the intervention of *C. perfringens* further destroyed the stability of the intestinal microbial composition, resulting in a large difference in bacterial species between days 19 and 13. Interestingly, the ileal microbial community in the *L. fermentum*-supplemented group was more similar to that of the CTR group at the phylum level both on days 13 and 19. This suggested that the remission of *L. fermentum* on gut health was related to the changes of microbial flora.

At the genus level, on day 13, coccidial infection increased the *Escherichia*–*Shigella* and *Lactobacillus* abundance in the ileum, and decreased the abundance of *Romboutsia* and *Candidatus_Arthromitus*, which were rescued by dietary lactobacillus intervention. Meanwhile, the *Candidatus_Arthromitus* was a biomarker genus in the *L. fermentum* addition group. According to studies, *Escherichia*–*Shigella* is a regular pathogen in *Enterobacteriaceae* that probiotic treatment is effective in decreasing [47,53]. Our current results show that two *Lactobacilli* reduced the abundance of *Escherichia*–*Shigella* in challenged broilers on day 13, which helped to reduce the intestinal inflammation. To the contrary, *C. perfringens* infection reduced *Escherichia*–*Shigella* richness on day 19. A possible explanation was that *C. perfringens*, also a harmful bacterium, competed with *Escherichia*–*Shigella* for nutrients, ultimately inhibiting its proliferation in the intestine. *Lactobacillus* is a well-known beneficial genus in the gut of animals and plays an important role in improving immunity, facilitating the absorption of nutrition, and maintaining gut integrity by promoting intestinal microbiota homeostasis [54]. Coccidial challenge increased ileal *Lactobacillus* abundance on day 13 and *C. perfringens* infection decreased it on day 19. As expected, *L. fermentum* and *L. paracasei* strikingly increased *Lactobacillus* abundance on day 19. Similarly, Song et al. also found that the increase in *Lactobacillus* was coupled to the relief of intestinal damage in broiler chickens with necrotic enteritis [13].

*Romboutsia* is a Gram-positive coccus that is reported to be predominant in healthy human mucosa and can produce acetic acid, formic acid, and lactic acid through breaking down monosaccharides and disaccharides [55]. Acetic acid is a short-chain fatty acid, which help maintain intestinal health. Therefore, the elevated *Romboutsia* by coccidial challenge on day 13 may be a feedback to fight against parasite infection, and this speculation could be strengthened by the association between increased *Romboutsia* and decreased inflammatory cytokine production in serum [56,57]. In addition, *Candidatus_Arthromitus* is a potentially beneficial bacteria for the development of mucosa and immune system in intestines and it can specifically induce T cell response [58,59]. Thus, at 13 days of age, the increased *Candidatus_Arthromitus* abundance in lactobacillus-supplemented groups, especially in the *L. fermentum*-supplemented group, might have enhanced the development of birds’ immune competence. Consistently, dietary *L. fermentum* also increased the population of *Candidatus_Arthromitus* on day 19. It is worthwhile to further explore the effect of *Candidatus_Arthromitus* on remitting intestinal damage in broilers with NE. These data illustrated that *L. fermentum* and *L. paracasei* exerted positive effects on intestinal microbiota, which may be used as an additive to promote intestinal health instead of antibiotics in broilers post-challenge. Moreover, our results may also provide an understanding for the use of *Lactobacilli* to alleviate chronic intestinal inflammation in patients.

## 5. Conclusions

Combined challenge with coccidia and *C. perfringens* impaired intestinal health in broilers by diminishing the proliferation of intestinal stem cells, inducing inflammatory responses, and disrupting intestinal microbiota structure. Dietary *L. fermentum* and *L. paracasei* supplementation attenuated intestinal damage through modulating JAK/STAT signaling and maintaining intestinal microbial homeostasis. Particularly, *L. fermentum* restored the proliferation of intestinal stem cells to some extent.

## Figures and Tables

**Table 1:** The feed ingredient composition and nutritional level (air-dried basis).

Ingredient, %	Days 0–19	Nutrient and Energy Composition ^3^	Days 0–19
Wheat	68.69	ME, kcal/kg	2930
Soybean meal	20.32	Crude protein, %	21.61
Fish meal	5.00	Lysine, %	1.16
Soybean oil	2.50	Methionine, %	0.60
CaHPO_4_	1.20	Methionine + Cystine, %	0.91
Stone powder	1.10	Calcium, %	1.13
NaCl	0.35	Available phosphorus, %	0.48
DL-Met	0.26	Threonine, %	0.75
Choline chloride, 50%	0.20	Sodium, %	0.14
Mineral premix ^1^	0.20		
L-Lys HCl, 78%	0.15		
Vitamin premix ^2^	0.03		
Total	100.00		

^1^ Trace element premix (provided per kilogram of feed) included the following substances: Cu, 8 mg; Zn, 75 mg; Fe, 80 mg; Mn, 100 mg; selenium, 0.15 mg; iodine, 0.35 mg. ^2^ Vitamin premix (provided per kilogram of feed) included the following substances: vitamin A, 12,500 IU; vitamin D_3_, 2500 IU; vitamin E, 18.75 mg; vitamin K_3_, 2.65 mg; vitamin B_1_, 2 mg; vitamin B_2_, 6 mg; vitamin B_12_, 0.025 mg; biotin, 0.0325 mg; folic acid, 1.25 mg; nicotinic acid, 50 mg; pantothenic acid, 12 mg. ^3^ Calculated values.

**Table 2:** List of gene primer sequences.

Gene Name	Accession Number	Primer Sequence (5′–3′)	Product Size [25]
*Lgr5*	XM_205518.1	CCTTTATCAGCCCAGAAGTGA	136
		TGGAACAAATGCTACGGATG	
*Znrf3*	M_015275473.1	GCCTCTACCAAGCCCAATCT	130
		GGTCGTCGGAAGTTGTGAG	
*Olfm4*	NM_001040463.1	GACTGGCTCTCTGGATGACC	108
		AGCGTTGTGGCTATCACTTG	
*Hopx*	NM_204556.1	GCAAGGTGAACAAGCATCC	227
		CCCAAGTAAACCCACTCTGAA	
*cdxA*	NM_204676.2	CAGTGAGTGTCCCCCATGTC	92
		GGGACAGATGTCTGCAGGTC	
*cdxB*	NM_204614.1	ATCTGGTTCCAGAATCGCCG	141
		TGGTGGGAACAGGGAACTTG	
*IL-1β*	NM_204524	ACTGGGCATCAAGGGCTA	131
		GGTAGAAGATGAAGCGGGTC	
*iNOS*	U46505	CAGCTGATTGGGTGTGGAT	158
		TTTCTTTGGCCTACGGGTC	
*TNF-α*	NM_204267	GAGCGTTGACTTGGCTGTC	64
		AAGCAACAACCAGCTATGCAC	
*IFN-γ*	NM_205149.1	AGCTGACGGTGGACCTATTATT	259
		GGCTTTGCGCTGGATTC	
*TGF-β4*	M31160	CGGGACGGATGAGAAGAAC	258
		CGGCCCACGTAGTAAATGAT	
*IL-13*	AJ621735	CCAGGGCATCCAGAAGC	256
		CAGTGCCGGCAAGAAGTT	
*JAK1*	XM_015290965.1	TGCACCGTGACTTAGCAGCAAG	168
		TCTGAATCAAGCATTCTGGAGCATACC	
*JAK2*	XM_015280061.1	TCGCTATGGCATTATTCG	197
		GTGGGGTTTGGTCCTTTT	
*JAK3*	NM_204996	CAGCCCCAACCAGATGTC	106
		CCGCTTGATGCCTTTGTAG	
*STAT1*	XM_015289392.1	TAAAGAGGGAGCAATCAC	112
		ATCAGGGAAAGTAACAGC	
*STAT3*	NM_001030931	AGGGCCAGGTGTGAACTACT	98
		CCAGCCAGACCCAGAAAG	
*STAT5*	NM_204779	CCCACCCCCATTACAACA	114
		GCAGCAGCTCCTCCACAT	
*STAT6*	XM_015274736.1	GCAACCTCTACCCCAACA	127
		TCCCTTTCGCTTTCCACT	
*TYK2*	XM_427671	GCCCCATGCAGGAGGAAT	119
		CTTTGCCACAGCCAGAATCAC	
*TAK1*	XM_015284677	CCAGGAAACGGACAGCAGAG	135
		GGTTGGTCCCGAGGTAGTGA	
*SHP2*	NM_204968	ATGTTGGTGGAGGGGAGAA	108
		GGGGCTGCTTGAGTTGC	
*SOCS1*	NM_001137648	CTACTGGGGACCGCTGACC	117
		TTAACACTGATGGCAAAGAAACAA	
*NF-κB p65*	NM_205129	GTGTGAAGAAACGGGAACTG	203
		GGCACGGTTGTCATAGATGG	
		TGGTGGGAACAGGGAACTTG	
*Actin*	NM_205518	GAGAAATTGTGCGTGACATCA	152
		CCTGAACCTCTCATTGCCA	

*Lgr5*, leucine-rich repeat containing G protein-coupled receptor 5; *Znrf3*, zinc and ring finger 3; *Olfm4*, olfactomedin 4; *Hopx*, homeodomain-only protein X; *cdxA*, caudal- type homeobox 1; *cdxB*, caudal-type homeobox 4B; *TNF-α*, tumor necrosis factor-α; *IFN-γ*, interferon-γ; *IL*, interleukin; *TGF-β4*, transforming growth factor β4; *iNOS*, inducible nitric oxide synthase; *JAK*, Janus-activated kinase; *STAT*, signal transducer and activator of transcription; *TYK2*, tyrosine kinase 2; *TAK1*, transforming growth factor kinase 1; *SHP2*, src homology-2 domain-containing protein tyrosine phosphatase 2; *SOCS1*, suppressors of cytokine-signaling 1; *NF-κB*, nuclear factor κB.

**Table 3:** The alpha diversity of ileal microbiota in broiler chickens ^1^.

Items	CTR	CCP	LF_CCP	LP_CCP	SEM	*p* Values
Day 13						
Shannon	2.68 ^b^	2.88 ^b^	4.20 ^a^	3.17 ^ab^	0.20	0.028
Simpson	0.67 ^b^	0.64 ^b^	0.83 ^a^	0.69 ^ab^	0.03	0.067
Chao1	551.65	605.25	974.46	709.70	82.54	0.282
Ace	578.86	625.59	1015.19	727.72	84.09	0.260
Day 19						
Shannon	3.01	3.90	3.47	2.30	0.29	0.240
Simpson	0.66	0.73	0.67	0.61	0.04	0.934
Chao1	720.14 ^ab^	1001.97 ^a^	865.56 ^ab^	276.30 ^b^	108.35	0.004
Ace	682.06 ^ab^	1030.91 ^a^	899.93 ^a^	295.84 ^b^	105.17	0.001

^1^ Data are expressed as mean and pooled SEM on days 13 and 19, respectively. Data were derived from 8 chickens in each group. ^a,b^ Values within a row with different superscripts differ significantly at *p* < 0.05. CTR, unchallenged group; CCP, coccidia- and *Clostridium perfringens*-challenged group; LF_CCP, challenged group with dietary supplementation of *Limosilactobacillus fermentum*; LP_CCP, challenged group with dietary supplementation of *Lacticaseibacillus paracasei*.

## Data Availability

The raw data of 16S amplicon sequencing has been uploaded to the NCBI database and the accession number is PRJNA1033806. Other data that support the findings of this study are available upon request from the corresponding author.

## Abbreviations

*cdx*: caudal-type homeobox; *Hopx*, homeodomain-only protein X; *IFN*, interferon; *IL*, interleukin; *iNOS*, inducible nitric oxide synthase; ISC, intestinal stem cell; *JAK*, janus-activated kinase; *Lgr5*, leucine-rich repeat containing G protein-coupled receptor 5; NE, necrotic enteritis; *NF-κB*, nuclear factor-κB; *Olfm4*, olfactomedin 4; *SHP2*, src homology-2 domain-containing protein tyrosine phosphatase 2; *SOCS1*, suppressors of cytokine-signaling 1; *STAT*, signal transducer and activator of transcription; *TAK1*, transforming growth factor kinase 1; *TGF-β4*, transforming growth factor-β4; *TNF-α*, tumor necrosis factor-α; *TYK2*, tyrosine kinase 2; *Znrf3*, zinc and ring finger 3.
